# Towards Quantum-Secured Permissioned Blockchain: Signature, Consensus, and Logic

**DOI:** 10.3390/e21090887

**Published:** 2019-09-12

**Authors:** Xin Sun, Mirek Sopek, Quanlong Wang, Piotr Kulicki

**Affiliations:** 1 Institute of Logic and Cognition, Sun Yat-sen University, Guangzhou 510275, China; xin.sun.logic@gmail.com; 2 MakoLab SA, 91062 Łódź, Poland; sopek@makolab.com; 3 Department of Computer Science, University of Oxford, Oxford OX13QD, UK; quaang@cs.ox.ac.uk; 4 Department of the Foundations of Computer Science, Catholic University of Lublin, 20950 Lublin, Poland

**Keywords:** blockchain, quantum computing, consensus, digital signature, lottery

## Abstract

While Blockchain technology is universally considered as a significant technology for the near future, some of its pillars are under a threat of another thriving technology, Quantum Computing. In this paper, we propose important safeguard measures against this threat by developing a framework of a quantum-secured, permissioned blockchain called Logicontract (LC). LC adopts a digital signature scheme based on Quantum Key Distribution (QKD) mechanisms and a vote-based consensus algorithm to achieve consensus on the blockchain. The main contribution of this paper is in the development of: (1) unconditionally secure signature scheme for LC which makes it immune to the attack of quantum computers; (2) scalable consensus protocol used by LC; (3) logic-based scripting language for the creation of smart contracts on LC; (4) quantum-resistant lottery protocol which illustrates the power and usage of LC.

## 1. Introduction

A blockchain is a distributed, transparent, and append-only ledger of cryptographically linked units of data (blocks), which incorporates mechanisms for achieving consensus over the blocks of data in a large decentralized network of agents who do not trust each other. It is a ledger in the sense that the data entries stored on the blockchain can be considered as generalized transactions. It is a distributed system in the sense that all miners (the peers who are in charge of updating the ledger) have separated, identical copies of the ledger. A blockchain is permissionless if everyone can be a miner on its network of nodes, otherwise it is permissioned. One of the most prominent application of blockchain technology is to enable the creation and distribution of cryptocurrencies, such as Bitcoin [1]. Another important application is the implementation of smart contracts [2], which are enforceable, irrefutable agreements among mutually distrusting peers which do not imply a trusted third party as their affirmation and administration mechanism.

The power of quantum computers and the capabilities of existing quantum algorithms [3] represent a threat to most of the existing public-key cryptographic systems. The current predictions [4] assume that by 2026 the chance of the practical availability of quantum computers is about 15% and by 2031 the chance grows to 50%. As almost all existing blockchain implementations have very deep reliance on the public-key digital signatures and are used for the transfer of value, they are particularly vulnerable to the attack of quantum computers. As it is pointed out by Fedorov et al. [5], blockchain technology as we know it today, may founder unless it integrates quantum technologies. While there are some interim solutions that incorporate post-quantum cryptography [6,7,8], they do not guarantee unconditionally secure solutions to the threat.

There is a considerable amount of research related to the quantum-safe blockchain [9,10,11] which could withstand attacks powered by forthcoming quantum computers. One of the most prominent proposals is the quantum-secured blockchain (QB) developed by Kiktenko et al. [9]. Due to the application of unconditionally secure message authentication based on quantum key distribution methodology, QB is immune from the attacks of quantum computers.

However, the major limitation of QB is the consensus protocol it adopts. In QB, the classical Byzantine agreement protocol [12] is used to achieve consensus among nodes of the distributed ledger. Kiktenko et al. have noticed that the classical Byzantine agreement protocol has serious shortcomings for QB as it becomes exponentially data-intensive if a large number of cheating nodes are present. Therefore, further research on efficient consensus protocol is required. In this paper, we report our research on a new consensus protocol which exhibits only quadratic dependence of resources on the number of miners. Our proposed consensus protocol is a combination of an unconditionally secure signature scheme and the YAC (Yet Another Consensus) algorithm [13]. Using the unconditionally secure signature scheme and the scalable consensus protocol, we put forward a proposal for a new quantum-secured permissioned blockchain called Logicontract (LC). The script language and smart contracts for QB have not been developed yet. In the work reported here, we developed a toy script language for LC that shows the main concepts behind the future implementation of smart contracts for LC. To demonstrate the power and usage of LC, we have also developed a proof-of-concept in the form of quantum-resistant distributed lottery protocol, which in turn uses the conceptual smart contracts on LC.

In Section 2, we construct our signature scheme for LC. We embed this signature scheme into the YAC algorithm described in Section 3. In Section 4 and Section 5, we develop a simple logic-based script language for LC and realize the lottery protocol proof-of-concept. We discuss related work in Section 6 and conclude this paper in Section 7 with an outline of the future work.

## 2. Unconditionally Secure Signature

Digital signatures are widely used to ensure the identity of a signer, the authenticity of a message, and to guarantee that a message is transferable. To react to the threat of quantum computers, unconditionally secure signature (USS) schemes have been proposed in recent years [14,15,16,17,18,19,20,21,22,23]. The new USS scheme proposed recently by Amiri et al. [23] has been proven to exhibit many advantages over the other existing USS schemes. The scheme assumes a message authentication code as a base for its operations. In this paper, we use the Toeplitz hash message authentication code as the base of the USS scheme and obtained a signature scheme useful in the permissioned blockchain.

### 2.1. Toeplitz Hash Message Authentication Code

Unconditionally secure authentication schemes have been extensively studied in the literature [24,25,26,27,28,29,30]. Carter and Wegman [24,25] were the first to construct authentication codes using hash functions. They also showed that one-time pad encryption can be used in combination with hash functions to construct unconditionally secure authentication schemes. This approach was further studied by Brassard [26], Desmedt [27], and Krawczyk [28,29]. In this paper, we proposed the use of the unconditionally secure authentication scheme based on the Toeplitz hash and apply it as a basis for the digital signature useful in Quantum Blockchain.

Toeplitz hash was first introduced by Krawczyk [28,29] for message authentication in the symmetric key model. Such an authentication scheme uses the Toeplitz matrix, generated by the symmetric key shared by the sender and the receiver, to hash the message using matrix-vector multiplication. When combined with a one-time pad encryption of the hash value, this scheme gives unconditional security of the message transfer.

Formally, let the length of all messages and their hash tags be $l_{m}$ and $l_{h}$, respectively. Let a $l_{h}$-bit string *r* be the symmetric key. The authentication code of the $l_{m}$-bit message *x* is calculated according to

$$h\left(x\right)=T_{S}\left(x\right)⊕r$$,

where $T_{S}$ is an $l_{h}\times l_{m}$ Toeplitz matrix generated by a string of length $l_{m}+l_{h}-1$ and ⊕ is the bit-wise XOR operator. Toeplitz matrices are characterized by having fixed Boolean diagonals. That is, *M* is a Toeplitz matrix if M[i,j]=M[i+1,j+1] and all elements of *M* are Boolean. For example, the following is a Toeplitz matrix generated by S=0110010 with $l_{h}=3$ and $l_{m}=5$:

$$T_{0110010}=\left[\begin{array}{ccccc} 1 & 0 & 0 & 1 & 0\\ 1 & 1 & 0 & 0 & 1\\ 0 & 1 & 1 & 0 & 0 \end{array}\right].$$

Since the left-to-right diagonal is fixed in a Toeplitz matrix, it can be fully described by its first column and first row. Given an n+m−1-bit string *S*, we construct an n×m Toeplitz matrix *M* as follows:

1. We map the first *n* elements of *S* into the first column of *M*, starting from the bottom M[n,1] to the top M[1,1].
2. We map the last m−1 elements of *S* into the first row of *M*, starting from the left M[1,2] to the right M[1,m].

Note that Toeplitz hash works only for messages of fixed length. To directly apply Toeplitz hash for message authentication on Quantum Blockchain, we require the length of every message communicated on the quantum blockchain to be less than $l_{m}$. If the length of a message is strictly less than $l_{m}$, then we pad the message with a sequence of 0s to extend its length to $l_{m}$ before we produce its authentication code.

Once a Toeplitz matrix $T_{S}$ between two peers is established, it can be reused for multiple messages while the encryption key *r* can only be used once for every message. Therefore, a hash function is uniquely determined by the encryption key *r*, which is an $l_{h}$-bit string, after the Toeplitz matrix has been established. Both the string *S* and the encryption key *r* are established by quantum key distribution.

### 2.2. Toeplitz Group Signature

We assume that, in the LC Quantum Blockchain, each pair of nodes is connected by a classical channel. Those nodes are also connected by quantum channels which form a quantum key distribution (QKD) network [31,32,33,34,35]. There are several implementations of the QKD networks and each of them can work with LC. At the current stage, we do not have a special preference for a specific QKD network. We have based our research on the assumption of the existence of QKD networks which make unconditionally secure communication possible, but we do not go deep into the implementation details of QKD networks. We assume that each pair of nodes establishes a sequence of private keys using the QKD network. Those keys will be used for secure communication in the signature scheme we propose.

For the signature scheme we propose a new scheme that we called the Toeplitz Group Signature (TGS), which is a combination of Toeplitz hash message authentication and a simplified variant of the signature scheme proposed by Amiri et al. [23].

**Definition** **1** (Toeplitz Group Signature Scheme)**.** *The Toeplitz Group Signature scheme Q is a tuple {P,M,Σ,Sign,Ver}.*

*1*.  The set $P=\{P_{0},P_{1},\ldots ,P_{n}\}$ is the set of the communication participants including the signer $P_{0}$, and the n potential receivers $P_{1}$ to $P_{n}$. We assume that a proportion of δ participants are honest, where $\delta >\frac{3}{4}$;
*2*.  $M={\left\{0,1\right\}}^{l_{m}}$ is the set of possible messages of length $l_{m}$;
*3*.  $\Sigma ={\left\{0,1\right\}}^{n^{2}l_{h}}$ is the set of possible signatures of length $l_{m}$;
*4*.  *Sign:M→Σ is a function that takes a message m∈M and outputs a signature σ∈Σ. There are two stages for signing a message—the distribution stage and the signing stage:*
  *(a)*  *The distribution stage:*
    *i.*  The sender randomly generates a bit strings $r_{1,1},\ldots ,r_{n,n}$. The length of every $r_{i,j}$ is $l_{h}$. We use $f_{1,1},\ldots ,f_{n,n}$ to denote the Toeplitz hash functions determined by these strings;
    *ii.*  The sender securely sends $r_{i,1},\ldots ,r_{i,n}$ to each recipient $P_{i}$;
    *iii.*  Each receiver $P_{i}$ sends $r_{i,j}$ to every other receiver $P_{j}$;].
  *(b)*  The signing stage: The signature for the message m is defined as $Sign\left(m\right):=(f_{1,1}\left(m\right),\ldots ,f_{n,n}\left(m\right))$.
*5.*  Verify:M×Σ×P→{True,False} is a function that takes a message m, a signature σ, and a participant $P_{i}$ and returns a Boolean value based on the validity of the signature, as verified by the participant $P_{i}$.
  *Formally, upon receiving a message/signature pair (m,σ), where σ is a signature of the form $\;(t_{1,1},\ldots ,t_{n,n})$, the receiver $P_{i}$ calculates the following test:*
  $$T_{j,i}^{m}=\left\{\begin{array}{cc} 1 & if\;t_{j,i}=f_{j,i}\left(m\right)\\ 0 & otherwise \end{array}\right.,$$
  *and $Verify(m,\sigma ,P_{i})=True$ if*
  $$\sum_{j=1}^{n}T_{j,i}^{m}\geq \left(\frac{1}{2}+2\left(1-\delta \right)\right)n.$$
  *That is, participant $P_{i}$ accepts the signature when more than $(\frac{1}{2}+2\left(1-\delta \right))n$ of the tests are passed, where δ is the proportion of honest participants.*

Using proofs similar to those proposed by Amiri et al. [23], it is possible to prove that TGS scheme has the following security properties:

**Theorem** **1.** *The TGS scheme meets the following security requirements:*

- ***Unforgeability**: It is not possible for an adversary to create a valid signature with the probability higher than some negligible level;*
- ***Transferability**: If an honest receiver accepts a signature, then any other honest receiver would also accept the signature;*
- ***Non-repudiation**: It is not possible for a signer to repudiate a legitimate signature he has created with the probability higher than some negligible level.*

## 3. Quantum-Secured Consensus

According to Nguyen and Kim [36], the consensus protocols used in blockchain technology can be categorized into two main types. The first type are the proof-based consensus protocols, which is often used in permissionless blockchains. The most famous proof-based consensus protocol is proof-of-work (PoW) [1] used in Bitcoin. The second type is the vote-based consensus algorithm, which is often used in permissioned blockchains. The most influential vote-based consensus is offered by the Byzantine fault tolerance [37,38,39,40] algorithm. When designing LC, we have focused our attention on the algorithm called “Yet Another Consensus” (YAC). YAC is a practical consensus algorithm that is used to provide Byzantine fault tolerance for the Hyperledger Iroha Blockchain framework (https://cn.hyperledger.org/projects/iroha.). The consensus algorithm in LC is the Quantum-Secured YAC (QSYAC), which is a variant of the original YAC, where the TGS replaces the public-key signature.

There are two types of participants in QSYAC—clients and peers. Those participants are connected by a quantum key distribution network. The quantum network is used to distribute private keys between participants. Moreover, every two peers are connected by a classical channel. Every client and every peer are also connected by a classical channel. We assume for the channels to be synchronous—every message that is sent by a channel will be received by the recipient within a fixed span of time. We assume at least $\frac{3}{4}$ of all peers are honest.

QSYAC runs in rounds. In every round there is a specific proposing peer. Other peers are called voting peers. Assume {1,…,n} is the set of all peers. Then, for round *r*, the peer (rModn)+1 is the proposing peer and all other peers are voting peers. Clients generate transactions and send them to all peers. Formally, a client *s* forms a transaction Txs, the format of which is described in Section 4; signs it by TGS; and send it to all peers. In every round, the proposing peer takes a subset of transactions from all transactions it has received and creates a block proposal. The block proposal will be edited and validated by voting peers. Voting peers are responsible for validating and reaching agreement on transactions in proposals and storing the transactions into blocks to make the blockchain. Every peer maintains a local copy of the blockchain in order to create/validate block proposals.

### 3.1. The QSYAC Protocol

One round of QSYAC consists of the following three phases—the proposing phase, the voting phase, and the decision phase:

1. The proposing phase. After checking the validity of signatures of transactions, the proposing peer generates a block proposal and sends it to voting peers. The block proposal contains an ordered list of transactions that will potentially be added to the blockchain in this round. The proposal is signed by the TGS scheme with the proposing peer being the sender and all voting peers as receivers;
2. The voting phase. The proposal is sent to all voting peers. Voting peers enter the voting phase, during which they exchange votes across the network:
  (a) The voting peer calculates a verified proposal after it receives a proposal from the proposing peer. A verified proposal is a subset of transactions from the proposal, defined to be valid by the voting peer. The block, that is generated by a voting peer, consists of transactions from the verified proposal and the hash value (Here the hash function is a collision-resistant hash function, not a Toeplitz hash. We require the length of the hash value of this hash function to be the same as the length of hash value of the Toeplitz hash.) of the collection of those transactions;
  (b) The vote on the block generated by the voting peer is formed by a pair which contains the hash value of this block and the TGS of this hash value. In this TGS scheme, the voting peer is the sender and all other peers (including the proposing peer) are receivers;
  (c) When a peer votes for a block, it generates an order for all peers for the current round. The order is generated by a function that takes the hash value of the block as the input and returns an order for all peers as the output. An order function *f* is required to produce a uniformly distributed output. That is, for two orders of peers $OD_{1}=\left(O_{1},\ldots ,O_{n}\right)$ and $OD_{2}=\left(O_{1}^{\prime },\ldots ,O_{n}^{\prime }\right)$, it holds that $|\left\{x\in {\left\{0,1\right\}}^{l}:f\left(x\right)=OD_{1}\right\}\left|=\right|\left\{x\in {\left\{0,1\right\}}^{l}:f\left(x\right)=OD_{2}\right\}|$, where *l* is the length of the hash value and || calculates the cardinality of a set.
3. The decision phase. Votes for a block are sent to each peer in the specified order. Let the order be $\left(O_{1},\ldots ,O_{n}\right)$. Acceptance or rejection of a block is achieved by the following decision phase:
  (a) Let i=1;
  (b) All votes are sent to the peer $O_{i}$;
  (c) Supermajority is defined to be a number greater than $\frac{3}{4}$ of all peers in the network. When $O_{i}$ has collected a supermajority of votes for one block, this set of votes enables the creation of an accepting message for the block. $O_{i}$ broadcasts the accepting message to all peers. Every peer who receives this accepting message adds the block to its local blockchain, broadcasts the accepting message to all peers and this round ends.
    A rejecting message is created when Oi has not collected the supermajority of votes for any block. $O_{i}$ broadcasts the rejecting message in the same way as the accepting message. Every peer sends its vote to the peer $O_{i+1}$ if it receives a rejecting message or it did not receive an accepting message within a predefined waiting period.
    The decision procedure then continues with peer $O_{i+1}$;
  (d) If no accepting message is broadcast at the end the decision procedure, then this round ends with the block rejection and there is no update of the blockchain.

**Figure 1:**
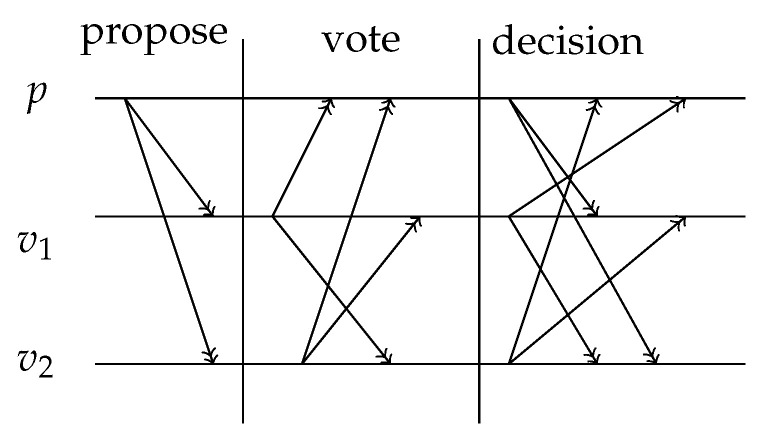
Quantum-Secured “Yet Another Consensus” algorithm (QSYAC).

A high-level visualization of QSYAC can be found in Figure 1, in which *p* is the proposing peer and $v_{1}$ and $v_{2}$ are voting peers.

### 3.2. Evaluation: Correctness, Scalability, and Security

#### 3.2.1. Correctness

The first part of the evaluation of the LC Quantum Blockchain is a proof that all honest peers keep the same copy of the blockchain, which is a corollary of the following theorems.

**Theorem** **2.** *If the proposing peer is honest in a round, then all honest peers will add the same block to its blockchain in this round.*

**Proof.**  Assume the proposing peer is honest, then all honest peers will receive the same message from the proposing peer. Then, all honest peers will generate the same order $\left(O_{1},\ldots ,O_{n}\right)$ and send their votes to $O_{1}$.

1. If $O_{1}$ is honest, then $O_{1}$ will collect a supermajority of votes, generate an accepting message, and send it to all peers. This means every honest peer will receive an accepting message and add the same block to its blockchain;
2. If $O_{1}$ is dishonest, since $O_{1}$ cannot counterfeit other peers’ vote, it cannot create a rejecting message. Now, another damaged $O_{1}$ can cause it to not send any message to other peers. There are two possible situations:
  (a) No honest peer receives an accepting message from $O_{1}$. Then after the pre-defined waiting period all honest peers will send their votes to $O_{2}$. As long as $O_{2}$ is honest, then all honest peers will make the same update of their blockchain.
  (b) Some honest peers receive an accepting message from $O_{1}$. Assume $O_{i}$ is an honest peer who receives an accepting message. Since $O_{i}$ will broadcast the accepting message, all honest peers will receive the accepting message.
  To sum up, all honest peers will receive an accepting message and add the same block to its blockchain even if $O_{1}$ is dishonest.

 □

**Theorem** **3.** *Assume the proposing peer is faulty in a round. If $O_{i}$ and $O_{j}$ are 2 honest peers, then it is impossible that they add different blocks to their blockchain in the same round.*

**Proof.**  Suppose in total there are 4f+1 peers, among which *f* peers are not honest and 3f+1 peer are honest. We prove the theorem by contradiction. Assume that $O_{i}$ and $O_{j}$ accept different blocks $B_{i}$ and $B_{j}$ in the same round. Then $O_{i}$ has received a supermajority of votes for $B_{i}$ and $O_{j}$ has received a supermajority of votes for $B_{j}$. That is, there are at least 3f+1 votes for $B_{i}$ and 3f+1 votes for $B_{j}$. This means that at least 2f peers have voted twice, which contradicts to the assumption that only *f* peers are not honest. □

#### 3.2.2. Scalability

The second part of LC evaluation is concerned with its scalability: If there are *n* peers, then the proposing peer sends n−1 message to the voting peers. The voting peers send at most $n^{2}$ messages in the decision procedure. Therefore, the communication complexity of QSYAC is $O(n^{2})$. This represents a quadratic dependence with respect to the number of peers, which is significantly slower than the exponential dependence of the classical Byzantine consensus. Therefore, QSYAC is a scalable protocol and, compared to the consensus protocol used in QB, definitely scales much better.

#### 3.2.3. Security

The third part of LC evaluation is concerned with its unconditional security. The unconditional security of TGS against forging, nontransferability, and repudiation makes LC secure in the era of quantum computers—even an adversary with unlimited computing power cannot forge an honest user’s digital signature. Therefore, the threat to Blockchain raised by quantum computers is resolved in LC.

## 4. Script Language and Smart Contracts for Logicontract

We are now introducing a toy scripting language for the smart contracts on LC. It shares some similarity with the script language of Bitcoin [41,42,43]. Scripts of transactions on LC are logical formulas ϕ which are built from arithmetic expressions *e* with the following syntax:

e::=x∣k∣e+e∣Hash(e)

ϕ::=e=e∣e>e∣Odd(e)∣After(e)∣¬ϕ∣ϕ∧ϕ.

Here, *x* is a variable with range over natural numbers and k∈N is a constant natural number. Hash is a collision-resistant hash function on natural numbers. Odd(e) means that *e* is an odd number. We also assume there exists a global clock. After(e) means that the time is later than *e* according to the global clock. ¬ and ∧ mean negation and conjunction, respectively.

While most existing blockchain platforms adopt procedural languages for expressing the contracts and reasoning about them, it has been argued that the logic-based languages could provide advantages over procedural languages [44,45]. For example:

- Logical contracts are often more compact than their procedural counterparts. This is because writing procedural contracts forces the programmer to write explicitly *what* has to be done and *how* to do it; while in the logical contracts the programmer only needs to write down what has to be done, without specifying how to achieve it.
- Writing contracts in a procedural language is error prone [46] since the order of instructions affects the correctness of the resulting contract, while logical contracts can be seen as a set of specifications, and the contracts are guaranteed to be correct with respect to the specifications.
- It is easier to formally verify a logical contract than to verify a procedural contract. To verify a procedural contract, a common technique is to construct a formal calculus with rigorous semantics and translate the procedural contract to expressions of the formal calculus [41,47]. Since logic by itself is a formal calculus, the verification of logical contracts is relatively easier.

Using the logic-based script language defined above, we now present the formal definition of the transaction in LC.

**Definition** **1** (transaction)**.** *A transaction T is a tuple (send,rece,sour,cert,prot), where*

- send is the sender of this transaction;
- rece is the set of receivers of this transaction and the amount of the currency they will receive. Formally, $rece=\{\left(r_{1},a_{1}\right),\ldots ,\left(r_{m},a_{m}\right)\}$;
- sour is the source, which is a list of transactions $\left(T_{1},\ldots ,T_{n}\right)$ to be redeemed by T;
- prot is the protection, which is a list of formulas. The number of formulas must be the same as the number of receivers. Formally, $prot=\{\left(r_{1},\phi_{1}\right),\ldots ,\left(r_{m},\phi_{m}\right)\}$. If a receiver $r_{i}$ wants to redeem T, then $r_{i}$ has to make $\phi_{i}$ true by providing appropriate certifications;
- cert is the certification, which is a list of valuation functions that map variables to natural numbers. The functionality of certifications is to satisfy the protection of the source transactions. The number of valuations must be the same as the number of the source transactions. Formally, $cert=\{\left(T_{1},V_{1}\right),\ldots ,\left(T_{n},V_{n}\right)\}$.

A transaction *T* redeems its sources if and only if the following holds:

1. *T* is properly signed by its sender;
2. The sender of *T* is one of the receivers in each of its source transactions;
3. The certification of *T* evaluates the protections of all its source to be true;
4. None of its source transaction has been redeemed.

We illustrate smart contracts on LC by a few examples.

**Figure 2:**
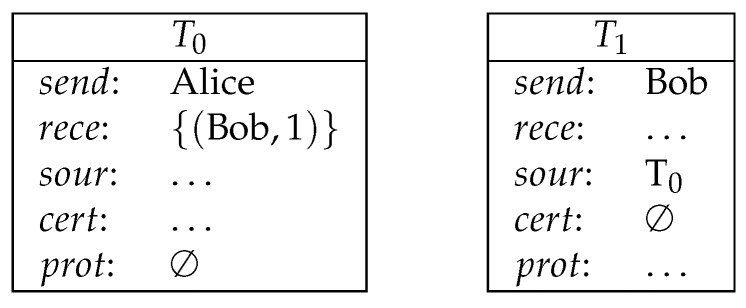
Direct payment.

**Example** **1** (direct payment)**.** *Alice pays 1 coin to Bob (see Figure 2).*

*Here, Alice creates the transaction T${}_{0}$, in which she sends 1 coin to Bob. Bob takes this coin by creating T${}_{1}$. The protection of T${}_{0}$ is empty. Therefore, Bob can redeem T${}_{0}$ as long as T${}_{1}$ is validly signed.*

**Figure 3:**
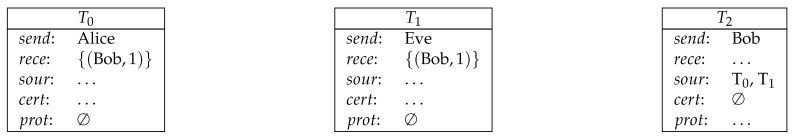
Payment from multiple sources.

**Example** **2** (payment from multiple sources)**.** *Alice pays 1 coin to Bob. Eve pays 1 coin to Bob (see Figure 3).*

*Here, Alice and Eve create transactions T${}_{0}$ and T${}_{1}$ to send coins to Bob. Bob takes these coins by creating T${}_{2}$, which redeems both T${}_{0}$ and T${}_{1}$.*

**Figure 4:**
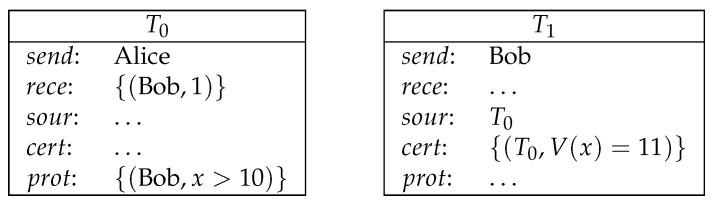
Conditional payment.

**Example** **3** (conditional payment)**.** *Alice pays 1 coin to Bob, on condition that Bob provides a number which is larger than 10 (see Figure 4).*

*Here, the protection of T${}_{0}$ is no longer empty. In order to redeem T${}_{0}$, Bob must provide a certification in T${}_{1}$ to satisfy the protection of T${}_{0}$.*

**Figure 5:**
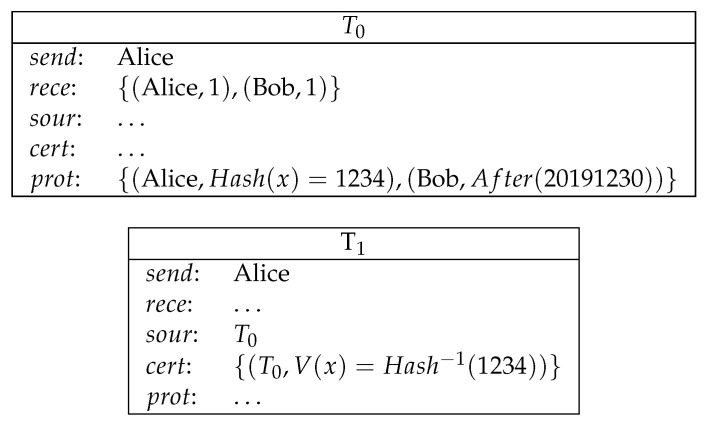
Commitment.

**Example** **4** (commitment)**.** *Alice commits a secret number x to Bob. Her secret number has a hash value 1234. Alice makes a deposit of 1 coin to Bob for her secret. If Alice reveals this secret before the time 20191230, then she can redeem her deposit. Otherwise Bob can redeem her secret (see Figure 5).*

## 5. Application: A Lottery Protocol on Logicontract

Now, we are ready to propose the concept of a distributed lottery on LC. Lottery is an important component of the multi-billion dollar gambling industry [48]. In general, in a lottery, there is an authority and a number of players. Players buy tickets to participate the game. Then, a random process is used to determine the winning tickets. In many lotteries, the revenue is huge and so there is an incentive for cheating. In order to ensure fair play and trust of the players, the ideal lottery protocol [49,50,51,52,53,54] should satisfy the following requirements:

1. Randomness. All tickets are equally likely to win;
2. Unpredictability. No player can predict the winning ticket;
3. Unforgeability. Tickets cannot be forged. Especially, it is impossible to create a winning ticket after the outcome of the random process is known;
4. Verifiablity. The number and the revenue of winning tickets are publicly verifiable;
5. Decentralization. The random process does not rely on a single authority.

Lottery protocols that satisfy the above requirements already exist [49,53]. With the advent of the quantum computing technology, it is reasonable to further require lottery protocols to satisfy the final requirement:

6. Quantum resistance. Even an adversary with a realistic quantum computer cannot rig the lottery.

Although quantum coin flipping [55,56,57,58,59,60,61,62]—a specific form of lottery—has been proposed, only the randomness and the quantum resistance have been studied. Other properties of lottery have rarely been addressed in quantum coin flipping. Using LC as a platform, we design a lottery protocol which satisfies all the above requirements.

**Figure 6:**
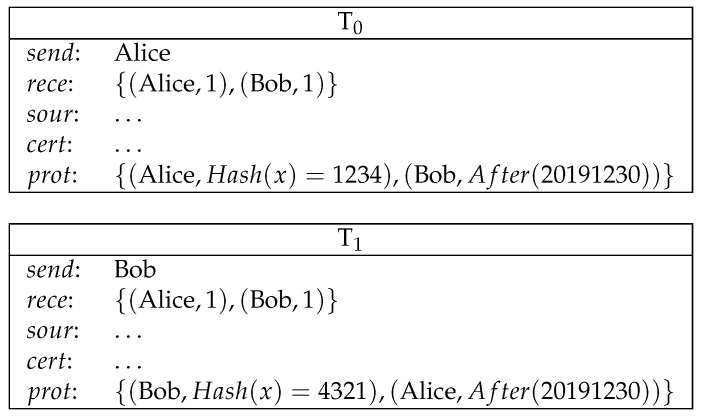
Lottery: step 1.

**Example** **5** (lottery)**.** *1. Alice commits a secret to Bob by making a deposit. Bob commits a secret to Alice by making a deposit (see Figure 6).*

**Figure 7:**
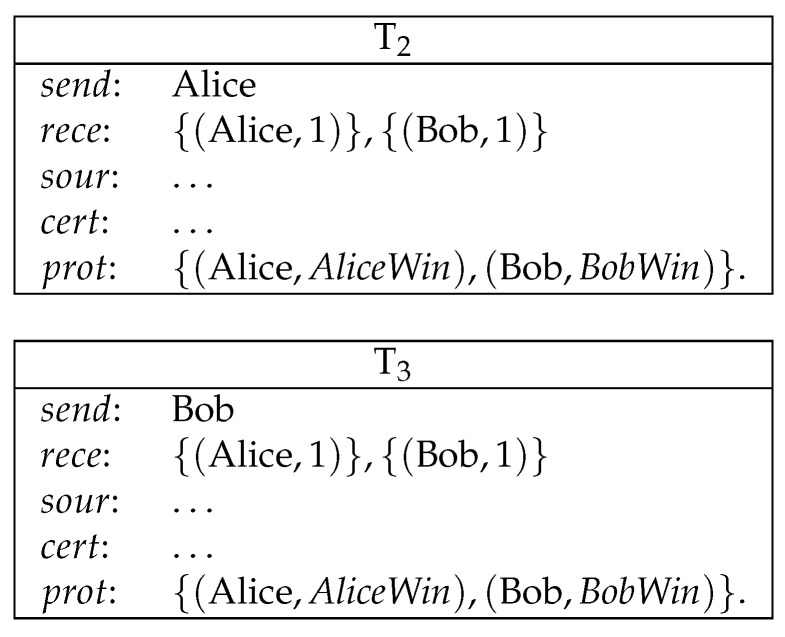
Lottery: step 2.

**Figure 8:**
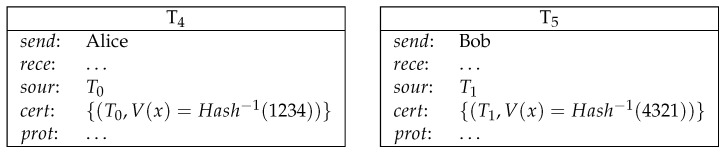
Lottery: step 3.

**Figure 9:**
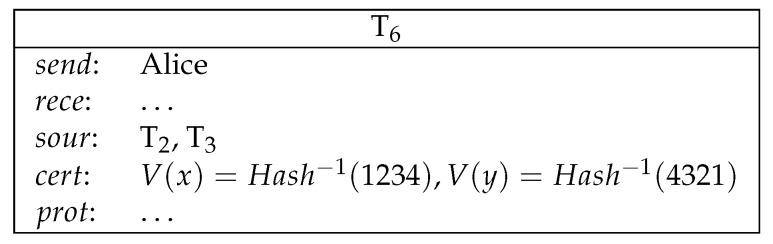
Lottery: final step, in case Alice is the winner.

**Figure 10:**
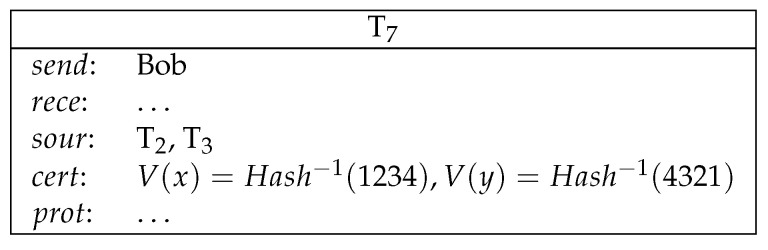
Lottery: final step, in case Bob is the winner.

2. Alice sends a conditional transfer to Alice and Bob. Bob sends a conditional transfer to Alice and Bob (see Figure 7).
  Here, AliceWin is specified by $\left(Hash^{-1}\left(x\right)=1234\right)\wedge \left(Hash^{-1}\left(y\right)=4321\right)\wedge \left(\left(Odd\left(x\right)\wedge Odd\left(y\right)\right)\vee \left(\neg Odd\left(x\right)\wedge \neg Odd\left(y\right)\right)\right)$. BobWin is specified by $\left(Hash^{-1}\left(x\right)=1234\right)\wedge \left(Hash^{-1}\left(y\right)=4321\right)\wedge \left(\left(Odd\left(x\right)\wedge \neg Odd\left(y\right)\right)\vee \left(\neg Odd\left(x\right)\wedge Odd\left(y\right)\right)\right)$.
3. Alice reveals her secret and gets her deposit back. Bob reveals his secret and gets his deposit back (see Figure 8).
4. Now both Alice and Bob’s secrets are public and the winner can be determined. The winner redeems the loser’s conditional transfer and his/her own conditional transfer. If Alice is the winner, then she redeems $T_{2}$ and $T_{3}$ (see Figure 9).
  If Bob is the winner, then he redeems $T_{2}$ and $T_{3}$ (see Figure 10).

## 6. Related Work

In order to protect Blockchain from an attack of quantum computers some efforts have been invested in recent years by several researchers. In general, there are two approaches adopted for this domain of research: quantum-resistant Blockchain and quantum-secured Blockchain.

On the one hand, in the proposals of quantum-resistant Blockchain [6,7,8], post-quantum signatures are used to replace classical signatures. In [7,8], the authors design a new lattice-based signature scheme to protect transactions on a blockchain. In [6], the authors propose a commit–delay–reveal protocol for the secure transition from Bitcoin’s current signature scheme to a quantum-resistant signature scheme. These solutions are based on the (unproven) assumption that certain computational problems cannot be efficiently solved by quantum computers. They do not offer unconditional security. On the other hand, quantum-secured Blockchain [5,9] do offer unconditional security by applying Quantum Key Distribution. Since QKD requires a fixed network of participants while quantum-resistant signature schemes do not have this requirement, it seems to us that in the near future quantum-resistant Blockchain can be used to replace the existing permissionless Blockchain, while quantum-secured Blockchain can be used to replace the existing permissioned Blockchain.

## 7. Conclusions and Future Work

In this paper, we described the development of Logicontract, a quantum-secured permissioned blockchain. LC adopts an unconditionally secure signature scheme based on Quantum Key Distribution and a vote-based consensus algorithm to achieve consensus on the blockchain. The advantage of LC compared to the existing quantum-secured blockchain is in the consensus protocol, which scales better with the number of peers.

We also presented a toy script language for LC and, as a proof-of-concept, designed a conceptual implementation for the quantum-resistant lottery protocol on LC. This lottery protocol satisfies many desired properties of the digital lottery including randomness, unpredictability, unforgeability, verifiability, and decentralization.

The quantum technology used in LC is the Quantum Key Distribution. QKD is a relatively matured technology which has been realized both by many research laboratories and by commercial companies (for example by ID Quantique [63]). Therefore, we conclude that LC can be practically realized by currently available technologies.

In our future research, we will systematically create the logic-based programming language for smart contracts on LC. There are some known disadvantages of the logic-based programming language. For example, it is difficult to estimate the cost of resources and the execution time of logically specified smart contracts. How to overcome these disadvantages is left to the future work. Despite the difficulties, our intent is to design smart contracts on LC which could solve the more complicated problems of modern financial industries. The formalism of smart contracts presented in this paper is similar to the simple smart contracts on Bitcoin’s blockchain. It is well-known that the script language of Bitcoin is not Turing-complete, which means that there are computations that cannot be realized by smart contracts on its blockchain. To overcome this limitation, in the future, we will develop a more powerful Ethereum-like formalism of smart contracts such that almost all distributed computing tasks can be realized by smart contracts on LC. In this paper, we analyzed LC from a theoretical perspective. In the future, we will run some experiments to study the security, scalability, and applicability of LC.
